# ‘We’re not all the same’—how heterogeneity among smallholder tree-crop farmers in Ghana generates different degrees of food insecurity

**DOI:** 10.1080/27685241.2025.2508143

**Published:** 2025-05-28

**Authors:** Martha Ataa-Asantewaa, Mirjam A. F. Ros-Tonen, Bart de Steenhuijsen Piters, Joyeeta Gupta

**Affiliations:** a Department of Geography, Planning and International Development Studies/Amsterdam Institute for Social Science Research (AISSR), University of Amsterdam, Amsterdam, The Netherlands; b Wageningen Economic Research, International Policy Subdivision, Wageningen University, Wageningen, The Netherlands

**Keywords:** Smallholder tree-crop farmers, household food and nutrition security, cocoa, oil palm, Ghana

## Abstract

Agricultural policies promoting smallholder participation in global markets for high-value commodities assume benefits for household food and nutrition security (FNS). However, existing literature often overlooks differences among smallholders. Using surveys, life interviews, and focus groups, this study applies the Household Access Food Insecurity Scale and dietary diversity scores to examine how household heterogeneity among Ghanaian tree-crop farmers affects FNS. Beyond standard FNS dimensions, we incorporate food sovereignty aspects like autonomy, cultural preferences, and sustainability. Only 47% of households were food secure, with significantly higher rates among those growing multiple tree crops (58%) and lower rates among landless farmers (30%). Households dependent on a single tree crop and landless households experience seasonal food insecurity due to low incomes. Interestingly, even the most economically secure multiple tree-crop households do not always achieve better dietary diversity, as spending choices influence nutrient intake. Cultural preferences impact FNS, particularly for older generations, while younger generations exhibit shifting dietary trends, highlighting the importance of cultural and generational factors. Intercropping is key to future FNS, given the widespread conversion of food-crop lands to tree-crop production. However, intercropping becomes difficult as tree crops mature, and excessive agrochemical use threatens sustainability and food safety. These findings underscore the need to explore intercropping in oil palm plantations, promote livelihood diversification, and raise awareness of more inclusive and sustainable farming practices. Future FNS research, policy, and practice must account for household heterogeneity and specific production contexts.

## 1. Introduction

Using World Census of Agriculture (WCA) data covering 80% of farms worldwide, Lowder et al. (2016) found that farms under 2 ha constitute 84% of all farms globally. Together, they produce 30–34% of the world’s food supply (Ricciardi et al., 2018). Earlier claims that farms under 2 ha in sub-Saharan Africa produce an estimated 80% of the continent’s food (FAO, 2017; GRAIN, 2014) have been debunked. However, Lowder et al. (2021) estimate that these farms still contribute about 30% of food commodities. Despite their importance in food provision, many smallholders, particularly in sub-Saharan Africa, are poor, food insecure, and lack access to markets and services (FAO, 2022; Rapsomanikis, 2015). According to FAO (2024), 58% of sub-Saharan Africa’s population faced moderate to severe food insecurity in 2023—double the global average of 29%— many of whom are smallholders (Frelat et al., 2016; Sibhatu et al., 2017). In Ghana, this figure was 42.4% (FAO, 2024).

Mainstream interventions promote market-based approaches, linking smallholders to markets and value chains (Tobin et al., 2016; Woodhill et al., 2012). Crop commodification is seen as a pathway from low-productive subsistence to high-productive market-oriented farming (Gereffi & Fernandez-Stark, 2016; Ros-Tonen et al., 2015), transforming smallholders into petty commodity producers (McMichael, 2013; Moyo, 2016; Olofsson, 2020). This shift reduces food crop production for household consumption.

Literature highlights the positive effects of value-chain integration on food and nutrition security (FNS) (Bonuedi et al., 2022; Legesse et al., 2023; Mango et al., 2016). However, growing evidence reveals smallholder heterogeneity (Bymolt et al., 2018; Jelsma et al., 2017; Peck et al., 2013; Perea et al., 2014; Signorelli, 2016), suggesting uneven impacts (Gebru et al., 2022; Kissoly et al., 2017). Moreover, increased income does not always improve FNS (Betge et al., 2020; van Westen et al., 2019). This paper aims to clarify how smallholders’ diverse characteristics and engagement in Ghana’s tree-crop sector affect households’ FNS. In doing so, we combine FNS and food sovereignty dimensions for deeper insights into factors that determine food safety and access to culturally appropriate food, such as autonomy over production and marketing and incomes and sustainable production (see Section 2).

This paper fills three key knowledge gaps. First, it nuances the dominant positive narrative of value-chain integration by offering insights into the impact of smallholder heterogeneity on FNS. Second, it integrates food sovereignty dimensions into the analysis to address the absence of indicators for food safety and preferences in the standard definition of FNS. Third, it adds a farmer-centred perspective (van Ewijk et al., 2024) rarely seen in existing FNS analyses.

The next section details the methodology, followed by the results on FNS across farmer profiles. We then show how incorporating autonomy and sustainability as food sovereignty dimensions that smallholders consider important adds insights beyond FNS measures. The discussion considers the theoretical and practical implications before presenting the conclusions.

## 2. Concepts and analytical framework

We define FNS as a situation in which “all people, at all times, have physical and economic access to sufficient safe and nutritious food that meets their dietary needs and food preferences for an active and healthy life” (FAO, 2008, p. 1). It encompasses four dimensions (FAO et al., 2023):

- *Availability*: the presence of food through one’s own production, markets, or other sources;
- *Access*: the physical and economic ability to obtain enough food;
- *Utilisation*: nutritional quality and sufficiency of food intake; and
- *Stability*: consistent availability, access and utilisation over time, avoiding acute, chronic or seasonal food insecurity (the focus of this paper).

We also include the more political and prescriptive food sovereignty concept, defined as:

“the right of peoples to healthy and culturally appropriate food produced through ecologically sound and sustainable methods, and their right to define their own food and agriculture systems. It puts those who produce, distribute and consume food at the heart of food systems and policies rather than the demands of markets and corporations” (Via Campesina, 2007).

Dimensions of food sovereignty include affordable food as a basic right, local production and consumption cycles, sustainability, autonomy over production, and appreciation of local knowledge and practices (Altieri, 2009; Clapp, 2014; Hopma & Woods, 2014; Jarosz, 2014; Wendimu et al., 2018). Our focus is on autonomy (self-determination of production, marketing, and consumption) and sustainability (responsible use of water, land and agrochemicals). Farmers identified these as their primary concerns (Ataa-Asantewaa, 2023). Moreover, they relate to food safety and food preferences – elements in the mainstream FNS definition that are poorly operationalised and rarely captured in food security studies (Manikas et al., 2023). Autonomy is a prerequisite for food preferences, while sustainability, particularly responsible agrochemical use, underpins food safety.

Food security and sovereignty differ across household profiles. A growing body of research challenges the notion of smallholders as a homogenous group. Differences exist in land size, gender, household demographics, access to land resources, market orientation, and livelihood diversification (Bymolt et al., 2018; Frelat et al., 2016; Hidayati et al., 2023; Jelsma et al., 2017; Laven & Ataa-Asantewaa, 2025; Olofsson, 2020). While few studies link farmer heterogeneity to FNS, available evidence suggests varying levels of food security (Bymolt et al., 2018).

**Figure 1.**
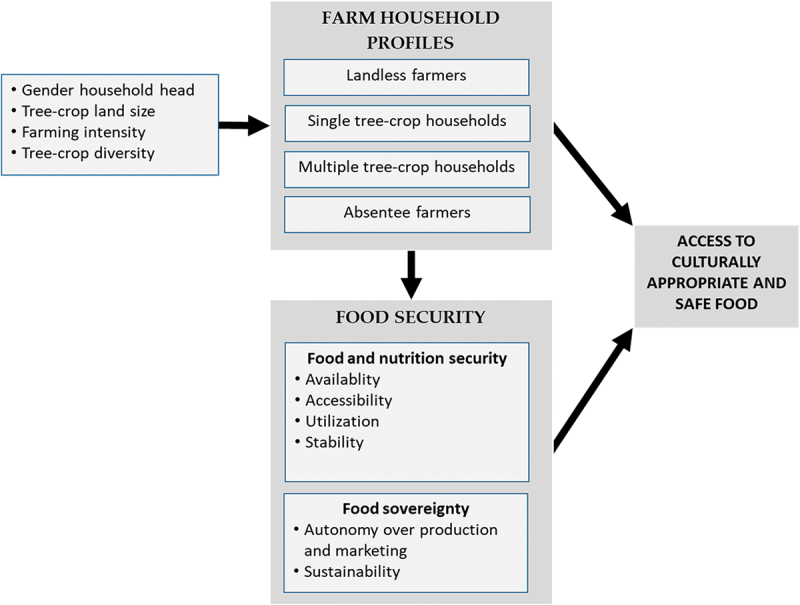
Conceptual scheme.

The conceptual scheme (Figure 1) that guided our analysis is based on the assumptions that (i) tree-crop smallholders are heterogeneous, shaped by gender, intensity of farming (part- or full-time), farm size, and diversity of tree crops grown, (ii) different farmer profiles experience different levels of food insecurity, and (iii) food security and sovereignty together influence access to culturally appropriate and safe food.

## 3. Materials and methods

### 3.1. Selection of the study sites

We selected cocoa and oil palm to analyse FNS among tree-crop farmers, as these are key agricultural commodities in Ghana and central to policies promoting smallholder market integration (MoFA, 2006, 2007, 2010). In 2023, Ghana produced 653,700 tons of cocoa, about 12% of global output. Though oil palm contributes less globally (2.56 million tons, <1% of global output) (https://www.fao.org/faostat/en/#data/), both are vital for rural livelihoods. Cocoa employs over one million farmers, with another million involved further down the value chain (https://cocobod.gh/). Oil palm employs an estimated three million people across its value chain (https://oilpalmresearch.org/).

**Figure 2.**
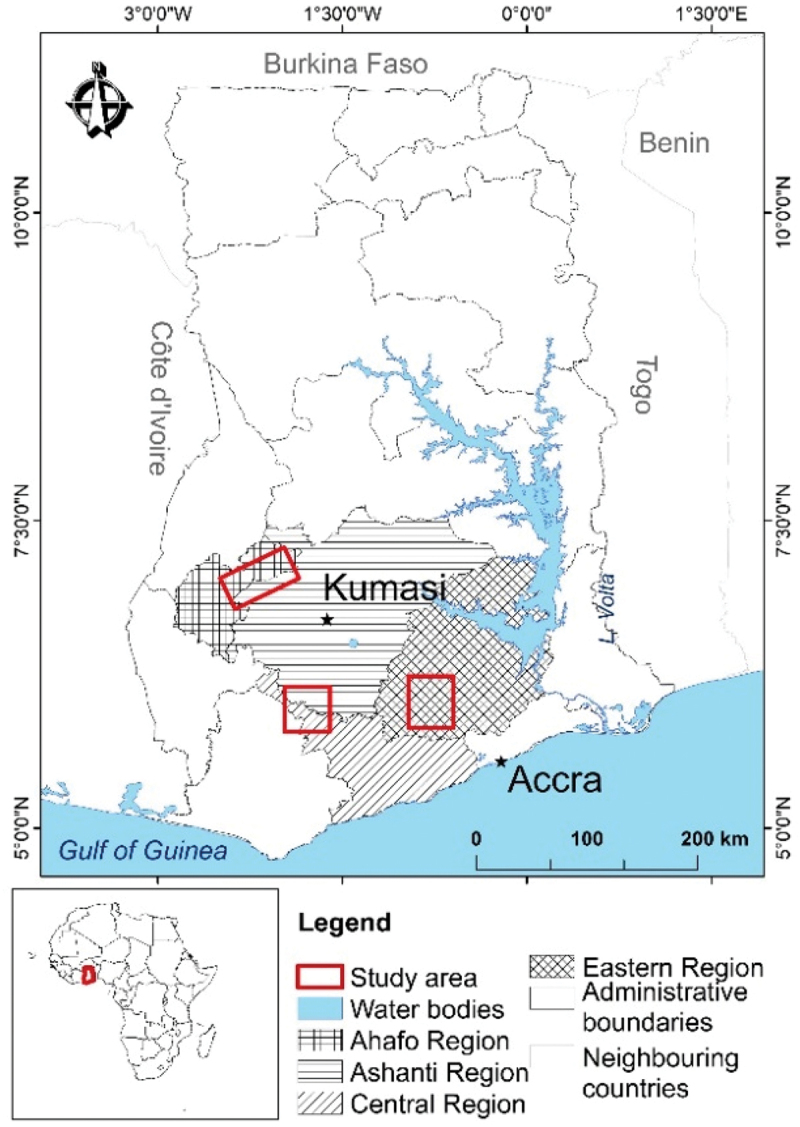
Location of the study sites.

Source: Prepared for the author by Kwabena Asubonteng, University for Development Studies, Tamale, Ghana.

The study was conducted in three regions (Figure 2): Kwaebibirem Municipal District (Kade area, Eastern Region, oil-palm dominant), Ahafo-North District (Tepa area, Ashanti Region, cocoa-dominant), and Upper Denkyira East Municipal District (Dunkwa area, Central Region) where cocoa is dominant but oil palm is promoted through the government’s oil palm master plan (MoFA , 2011) and the Twifo Oil Palm Plantations (TOPP) outgrower scheme. All sites are in Ghana’s semi-deciduous forest zone, which has a wet semi-equatorial climate with bimodal rainfall – a major rainy season (March – July) and a minor one (September – October). Temperatures range from 18–35°C (annual average: 26.5°C), and rainfall varies from 1,300–1,800 mm annually (Yamba et al., 2023).

Food crops in the area comprise cassava, cocoyam, plantain, and maize, which are harvested from August to November. Cocoa’s main harvest (70–80% of annual yield) is October-May, with a minor harvest June-September (Taylor et al, 2025Taylor et al. 2025). Oil palm is harvested year-round, peaking February – May, with a lean season during September- December (Khatun et al., 2020).

Communities were selected in consultation with relevant institutions, including the Department of Agriculture (Kade area), Armajaro Ghana Limited (Tepa area), and the TOPP scheme (Dunkwa area). The Kade and Tepa areas were assumed to be typical oil palm and cocoa areas, respectively (Asubonteng et al., 2018; Osei-Amponsah et al., 2012) and selected to achieve an equal representation of cocoa and oil palm farmers. However, few cultivated oil palm only; most also grew cocoa (Table 1).

Each study site was divided into three strata – north, centre, and south – to ensure a broad geographical spread. Two communities per stratum were purposefully selected based on accessibility and public transport availability (including taxi or tricycle). Two communities were only accessible by motorcycle.

**Table 1.** Study participants per site and crop.

Study site/tree crop	Kwaebibirem Municipal District, Eastern Region (Kade area) (*n*=51)	Ahafo Ano-North District, Ashanti Region (Tepa area) (*n*=60)	Upper Denkyirah East Municipal Central Region (Dunkwa area) (*n*=57)	Total
*Survey respondents*				
Cocoa	9	49	29	87 (51.8%)
Oil palm	5	0	3	8 (4.8%)
Both cocoa and oil palm	37	11	25	73 (43.4%)
Total of survey respondents per study site	51 (30.4%)	60 (35.7%	57 (33.9%)	168 (100%)
*Life interviews*	48	45	50	143
*Focus groups*	35	30	30	95

^a^The number of life interview respondents is lower than that of survey respondents because some had passed away in the interim (*n*=5), while others were unavailable due to travel (*n*=40), with the latter group consisting mainly of migrants.

*Source*: The authors.

### 3.2. Data collection methods

We conducted a cross-sectional survey among 168 tree-crop farming households across 18 communities between 2015 and 2017 during a period of neither food abundance nor scarcity (December-February). Despite the time lapse between data collection and the publication of this paper, the findings remain relevant, primarily because they highlight the heterogeneity among smallholders and its impact on their FNS and the importance of integrating FNS and food sovereignty dimensions in food security analysis.

Entry to communities followed customary approval from Chiefs and/or Assembly members, followed by a community meeting to explain the purpose of the research. To minimise bias, interviewers spread out in different directions in each community, visiting every household and interviewing available cocoa and/or oil palm farmers willing to participate after informed consent. Ethical principles of voluntary participation, confidentiality, transparency, and honesty were upheld.

We intentionally included female farm owners, who are often underrepresented in research, especially in the cocoa sector (Bymolt et al., 2018; Laven, 2010; Laven & Ataa-Asantewaa, 2025). We identified female smallholders – often living away from the farm plot or involved in non-farm activities, leaving farm management to caretakers – through networks of purchasing clerks and licenced buyers. Additional snowball sampling ensured a representative share of female-headed households (20%; *n* = 33), close to the 16% in a nationwide cocoa study (Bymolt et al., 2018). Additionally, 16 women from male-headed households participated, bringing female respondent representation to 30%. Moreover, spouses often contributed responses in interviews with male respondents.

Our sample size met cluster analysis requirements, following recommendations to have at least ten times more observations than variables (Mooi & Sarstedt, 2011), with each cluster holding ≥ 30 cases to allow for testing statistically significant differences among groups (Pallant, 2016; Stevens, 2009).

The survey covered household demographics, assets, land access, incomes and expenditure, investments in agriculture, market and non-market production, livestock, input use, access to markets, support services, and natural resources, experienced food insecurity, and dietary diversity. It also included questions on autonomy in decision-making over crop choice, buyer selections, and income use.

In July and early September 2018, six mixed-gender focus groups were held with 88 survey respondents to validate survey results. Invitations were made via community announcements and phone calls. Some deceased farmers were replaced by a spouse, relative or caretaker, bringing the total number of participants to 95.

Focus group participants used historical timelines to assess land and food source changes (see Supplementary Material 1 for the workshop protocol). Proportional piling of a fixed number of pebbles, beans or comparable items (Mariner & Paskin, 2000) was used to assess the relative importance of income sources, wealth, problems, and priorities. Seasonal calendars helped examine fluctuations in food production, availability, access, and stability. Discussions also addressed experienced food insecurity, coping strategies, and natural resource access. The cultural food preference and sustainability dimensions of food sovereignty (soil fertility, weed, pest and disease control, use of agrochemicals, farm waste management, water use, and seed sources) were also discussed in the focus groups. The autonomy dimension of food sovereignty was covered by survey questions on household control over decisions regarding crop choice for consumption and market, choosing a buyer, and spending of household income.

### 3.3. Data processing and analysis

*Food availability* was estimated using survey data on farmers’ food crop and livestock production and access to food stores, supermarkets, or markets within a 30-minute walk (2 km). *Food access* was assessed using indicators from the Household Food Insecurity Access Scale (HFIAS), a widely used experience-based measure alongside the Food Insecurity Experience Scale (FIES). We used HFIAS as it was designed for developing countries, whereas FIES is more suitable for global comparisons (Ballard et al., 2013).

**Figure 3.**
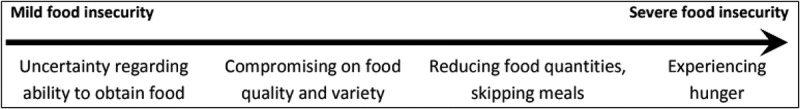
Household food insecurity along a severity continuum.

Source: Adapted from Ballard et al. (2013).

The HFIAS helps categorise households along a food insecurity severity scale from food secure to severely food insecure (Figure 3) through simple computations (Coates et al., 2007; Leroy et al., 2015). Though HFIAS includes nine questions across four domains, these can be adapted to cultural context if internal consistency and reliability (Cronbach alpha of ≥ 0.85) are maintained (Leroy et al., 2015). During pre-testing, respondents found several questions offensive, so we used one representative question per domain based on pilot testing:

- *Anxiety*: Did you ever worry that your household would not have enough food?
- *Dietary quality*: Were you or any household member not able to eat the kinds of foods you preferred because of a lack of resources?
- *Eating less*: Did you or any household member have to eat a smaller meal than you felt you needed because there was not enough food?
- *Going hungry*: Did you or any household member go a whole day and night without eating anything because there was not enough food?

These questions proved consistent and reliable, with a Cronbach alpha of 0.856 and a strong mean inter-item correlation of 0.6 (Hussein et al., 2018; Pallant, 2016). The questions referred to the 30 days prior to the interview, and responses were recorded as yes/no for occurrence and 1 (seldom; 1–2 times), 2 (occasionally; 3–10 times), or 3 (regularly; >10 times) for frequency when applicable. We computed the severity of food insecurity (Figure 3) per profile following the procedure outlined in Coates et al. (2007). Statistical significance was tested using Chi-square and Fisher’s exact tests – both of which are suitable for the categorical and scale variables used in this study (Pallant, 2016). The tests were conducted at a significance level of *p* ≤ 0.05, with adjusted residues of ± 2 indicating significant differences (Pallant, 2016).

*Food utilisation* was measured using the Household Dietary Diversity Score (HDDS), a proxy for diet quality (FANTA Food and Nutrition Technical Assistance Project, 2006; Kennedy et al., 2013; Leroy et al., 2015). The survey included 15 locally relevant food groups (e.g. staple foods like *fufu*, *ampesi*, *banku*, and jollof rice) as pre-testing revealed that farmers recalled their diets more accurately when asked about local dishes rather than individual food groups. During data entry, the 15 food groups were aggregated into the standard 12 food groups of the Food and Nutrition Technical Assistance II (FANTA) project (see Table 6 and Supplementary Material 2). Consumption of a food group was scored as 1, non-consumption as 0, and summed to create the HDDS (range 0–12). The mean HDDS of all households in a profile was the profile’s dietary diversity score. We recognise that diversity metrics vary by age and children (e.g. women of reproductive age vs children) (Leroy et al., 2015), but such distinctions were beyond the scope of this study.

*Food stability* focused on seasonal food insecurity. A survey question asked respondents to indicate months in which they struggled to access food.

Quantitative survey data was entered into Microsoft Excel for simple calculations and to transform certain variables for cluster analysis and basic statistics in the Statistical Package for Social Sciences (SPSS) (Pallant, 2016). To adjust for inflation, income data were adjusted to January 2017 values using Ghana’s consumer price index provided by the International Monetary Fund.

We applied a two-step cluster analysis to identify natural homogenous groupings in the data, aiming for high within-group homogeneity and between-group differences (Mooi & Sarstedt, 2011). This method handles both continuous and categorical variables, determines the optimal number of clusters, and evaluates the quality of the cluster solution using the silhouette value, which ranges from −1.0–0.2 (poor quality), 0.2–0.5 (fair quality), and 0.5–1.0 (good quality). Additionally, the analysis provides insights into variable importance and cluster size ratio (Blaikie, 2003; Mooi & Sarstedt, 2011). Supplementary Material 3 provides more details on the iterative clustering process and the variables used.

Notes from focus group discussions were transcribed by two assistants to ensure inter-observer reliability. Transcripts were coded and analysed in ATLAS.ti, using both deductive and inductive coding (Boeije, 2009). Seasonal calendar sheets developed in the focus groups were transposed into two-dimensional tables (time/activity) supported by accompanying notes to ensure legibility and presentability (see Supplementary Material 4).

## 4. Results

### 4.1. Farmer profiles

Cluster analysis revealed land ownership as the main distinguishing variable among tree-crop farming households, followed by tree-crop diversity, resulting in four distinct profiles with minimal overlap. These profiles, briefly characterised below, form the basis for analysing varying degrees of FNS. Basic characteristics per profile and associated statistics are presented in Supplementary Material 5.

*Profile 1*: Landless farming households (*n* = 30)

These are mainly migrant households with young heads (median age: 39) and large families (average: 7 members). All cultivate cocoa, either as sharecroppers or caretakers for absentee farm owners. None grow oil palm, hence their location in the cocoa-dominated Tepa and Dunkwa areas. The median gross annual income for this cluster is USD 556.80.

*Profile 2*: Single tree-crop households (*n* = 48)

Headed by older individuals (median age: 52), 21% female. They manage their own farms, almost exclusively growing cocoa (92%), which is why most (54.2%) live in the cocoa-dominated Tepa area. About 25% earn additional income by selling farm labour, but non-farm income is low (14%). Median gross annual income is USD 1,082.75.

*Profile 3*: Multiple tree-crop households (*n* = 64)

Mostly male-headed (89%), growing cocoa, oil palm, and occasionally citrus or cashew (26.6%). Heads are typically 55 years old, similar in age to Profile 2. Present across all study sites, though slightly more (52%) in the Kade area. They operate the largest farms (median size: 6 ha). The median gross annual income is USD 1,562.

*Profile 4*: Absentee households (*n* = 26)

Headed by significantly older individuals (median age: 70), with a relatively high proportion of female-headed households (35%). Most households in this group grow cocoa (65.4%), while the remaining 34.6% cultivate both cocoa and oil palm. Farms are run by caretakers, and households are evenly spread across the three study areas. The median gross annual income is USD 1,388.60.

### 4.2. Food and nutrition security across household profiles

#### 4.2.1. Food availability

Food availability was assessed through household production of food crops and livestock. Most households (*n* = 157; 94%) cultivated food crops, primarily plantain, cassava, cocoyam, vegetables, and maize. About 64% (*n* = 101) interplanted food crops within tree-crop farms; 20% (*n* = 31) grew them in dedicated food-crop farms (*mmaafuo* or women’s farms), and 16% (*n* = 25) combined the two).

Table 2 shows that the proportion of households growing food crops does not differ significantly across profiles, but key differences exist in *where* food is grown:

- Interplanting is significantly more common among landless farmers and less common among multiple tree-crop farmers. This is likely due to younger farms among sharecroppers, where intercropping is still possible, unlike mature farms that are more common among multiple tree-crop households.
- *Mmaafuo* (women’s farms) are more common among absentee households, whose tree-crop farms are managed by caretakers, while landless households rarely have dedicated food-crop farms due to limited land access.
- Mixing interplanting and *mmaafuo* is practised by around one-fifth of single and multiple tree-crop households but is far less common among landless and absentee households. The lower prevalence of this combination among landless and absentee households is due, respectively, to limited land access and disengagement from active tree-crop farming. The lower incidence of mixing interplanting and *mmaafuo* among landless farmers is statistically significant (Table 2).

**Table 2.** Tree-crop farmer households’ food production by source and profile.

Profile	Landless farming households (*n* = 30)^a^		Single tree-crop households (*n* = 48)^a^		Multiple tree-crop households (*n* = 64)^a^		Absentee households (*n* = 26)^a^		Total households (*N* = 168)	
Food production	n^b^	%	n^b^	%	n^b^	%	n^b^	%	N	%
Total growing food	30	100	46	95.8	58	90.6	23	88.5	157	93.5
Of which: Interplanting in tree-crop farm	27	90+	30	65.2	31	53.4-	13	56.5	101	64.3
Growing on the food-crop farm (*mmaafuo*)	2	6.7-	5	10.9	15	25.9	9	39.1+	31	19.8
Combination of inter-planting and *mmaafuo*	1	3.3-	11	23.9	12	20.7	1	4.3	25	15.9

^a^n refers to the total number of households per profile; ^b^ n refers to the number of households in the profile growing food; N refers to the total number of respondents. Chi-Square test where minimum cell frequency is 5 or higher; Fisher’s exact test where minimum cell frequency is less than 5. Test significant at *p* ≤ 0.05. * = statistically significant relationship based on a value of the adjusted residue (AR). Adjusted residue (AR) >+2 indicates a significantly higher proportion than expected and an adjusted residue (AR) < −2 indicates a significantly lower proportion than expected. P-value total growing food: 0.174; P=value place of growing food crops: 0.001 * .

*Source*: Survey, 2015; 2017.

In the 12 months before the survey, 81% (*n* = 136) harvested food crops. The most common were plantain (92%), cassava (83%), cocoyam (60%), vegetables (56%), and maize (30%). Few households mentioned rice (3.7%). Each profile harvested an average of three food crops, with no significant differences between the profiles (see Supplementary Material 6).

Of those who harvested food, 74% (*n* = 100) sold part of their produce, especially staple foods like plantain (72% of those who harvested plantain) and cassava (51% of those who harvested cassava), with no significant differences among profiles. This suggests sufficient food was available, though some sales may have been driven by the need to cover essential expenses such as healthcare, education, or unforeseen emergencies while food needs were unmet. However, we found no statistically significant differences in degrees of food insecurity based on the HFIAS scale between households growing or not growing food crops.

Most households (*n* = 121; 72%) also raised livestock, mainly small stock – chickens (76%), goats (54%), sheep (31%), and pigs (2.5%). Other livestock (cattle, donkeys) were rare (2.5%) and only found in multiple tree-crop and absentee households. Only sheep ownership varied significantly across profiles, being higher among landless households. This may relate to “keep & share” arrangements, where landless households rear poultry and small stock for others in exchange for a share of the proceeds.

In summary, households across all profiles had food and small stock available through their own production. However, landless households relied heavily on interplanting due to land constraints, while absentee farmers – freed from daily tree-crop farming after entrusting their tree-crop farms to caretakers – were more likely to cultivate food in *mmaafuo* plots.

#### 4.2.2. Food access

Food access was assessed in terms of physical proximity to food markets and economic capacity attributable to profile characteristics. *Physical* access was not a limiting factor; all 18 study communities had markets or provision stores within 2 km, where households commonly bought items like fish, meat, rice, noodles, salt, sugar, spices, drinks, and cooking oil. When farmers lack their own produce, they also purchase plantain, cassava, yams, vegetables, and other food crops.

Most multiple tree-crop farmers reported being able to buy food they do not produce themselves when needed. In contrast, significantly fewer landless households could do so (Table 3). Focus group discussions revealed that caretakers, in particular, relied on labour-for-food arrangements or foraging rather than market purchases. Meanwhile, better-off absentee and multiple tree-crop households could afford foods like noodles and “perfume rice” (aromatic processed rice, usually with jasmine).

**Table 3.** Households able to buy food items from the market when needed per profile.

Profile	Landless households (*n* = 30)		Single tree-crop households (*n*= 48)		Multiple tree-crop households (*n*= 64)		Absentee households (*n*=26)		Total households (*N*=168)		P-value
Access	n	%	n	%	n	%	n	%	N	%	
Economic access	9	30−	21	43.8	42	65.6+	14	58.3	86	51.2	0.008*

Chi-Square test, X^2^ (9, *n* = 168) = 11.865, Cramer’s V= 0.266, test significant at *p*≤0.05. *: *p* ≤ 0.008 is significant. +: adjusted residue (AR) >2; − adjusted residue (AR) < −2 determines which profiles show significance, with + indicating a significantly higher proportion than expected and - indicating a significantly lower proportion than expected.

indicated with + or–.

*Source*: Survey, 2015; 2017.

*Economic access* was measured using the Household Food Insecurity Access Scale (HFIAS; see Section 3.3). Over half of the landless, single tree crop, and absentee households reported some level of food insecurity in the 30 days before the interview, with statistically significant differences across profiles (*p* = 0.02). The percentage of food-secure households was significantly higher among multiple tree-crop households and significantly lower among landless households.

Severe food insecurity affected 7% of all households in the month preceding the survey, ranging from 3% among multiple tree-crop households to 13% among single tree-crop households (Table 4). The differences likely reflect variations in own food production and income to buy food. Multiple tree-crop and absentee households generally had greater purchasing power, giving them better access to food markets and, therefore, greater FNS compared to landless and single tree-crop households.

**Table 4.** Degree of household food insecurity access prevalence among household profiles.

Profile	Landless households (*n* =30)		Single tree-crop households (*n*=48)		Multiple tree-crop households (*n*= 64)		Absentee households (*n*=26)		Total households (*N* = 168)		*P*-value
Degree of food (in)security	n	%	n	%	n	%	n	%	N	%	
Food secure	9	30.0-	21	43.8	37	57.8+	12	46.2	79	47.0	0.02*
Mildly food insecure	14	46.7+	9	18.8-	21	32.8	9	34.6	53	31.5	
Moderately food insecure	4	13.3	12	25.0+	4	6.3-	4	15.4	24	14.3	
Severely food insecure	3	10.0	6	12.5	2	3.1	1	3.8	12	7.1	

Fisher’s exact test, Cramer’s V =0.195. Test significant level *p*≤0.05, *test significant.

+ indicates a significantly higher proportion than expected; - indicates a significantly lower proportion than expected.

*Source*: Survey, 2015; 2017.

#### 4.2.3. Food utilisation

Dietary diversity analysis showed that households consumed 8 of the 12 food groups within the 48 hours preceding the survey. Although there is no global standard for adequate dietary diversity, consumption from more than six food groups is generally seen as nutritionally sufficient (Ruel, 2003; Kennedy et al., 2013).^1^^1^It should be noted that dietary diversity scores assess nutritional quality based on the variety across different food groups, but do not measure the quantity of consumption or the specific macro- and micronutrients consumed. No significant differences in mean household dietary diversity scores were found across profiles, suggesting relatively similar diets. However, meat was consumed significantly more often by single tree-crop households than by landless ones (Table 5). This relative dietary similarity is likely due to households relying more on their own production than market purchases, reflected in the low consumption of items they do not produce, such as dairy products. Thus, income levels did not appear to influence diets at the time of the survey, as higher incomes were often spent on school fees and other expenses.

**Table 5.** Household consumption of 12 food groups by profile.

Profile	Landless households (*n* =30)		Single tree-crop households (*n*=48)		Multiple tree-crop households (*n*= 64)		Absentee households (*n*=26)		Total households (*N* = 168)		P-value
Crops	n	%	n	%	n	%	n	%	N	%	
Cereals	25	83.3	38	79.2	48	75.0	18	69.2	129	76.8	0.610
Root & tubers	28	93.3	46	95.8	60	93.8	24	92.3	158	94.0	0.884
Vegetables	29	96.7	46	95.8	61	95.3	25	96.2	161	95.8	1.000
Fruits	21	70.0	32	66.7	40	62.5	11	42.3−	104	61.9	0.136
Meat	5	16.7−	28	58.3+	32	50.4	10	38.5	75	44.6	0.003*
Fish	28	93.3	40	83.3	57	89.1	23	88.5	148	88.1	0.650
Eggs	16	53.3	31	64.6	32	50.0	9	34.6−	88	52.4	0.097
Legumes	25	83.3	31	64.6	43	67.2	17	65.4	116	69.0	0.311
Dairy	8	26.7	16	33.3	16	25.0	5	19.2	45	26.8	0.591
Sugars	21	70.0	36	75.0	39	60.0	18	69.2	114	67.9	0.455
Oils	25	83.3	39	81.3	53	82.8	18	69.2	135	80.4	0.480
Condiments	16	53.3	22	45.8	25	39.1	14	53.8	77	45.8	0.469

Chi-Square test where minimum cell frequency is five or higher; Fisher’s exact test where minimum cell frequency is less than 5. Test significant at *p*≤0.05 * = statistically significant relationship indicated with a value of adjusted residue. Adjusted residue (AR) >+2 indicates a significantly higher proportion than expected; adjusted residue (AR) < −2 indicates a significantly lower proportion than expected.

*Source*: Survey, 2015; 2017.

Focus group discussions added nuance. Landless farmers mainly depend on their own crops, labour-for-food arrangements, and wild foods rather than markets due to limited land and moderate incomes (see Supplementary Material 7). In contrast, “entrepreneurial farmers” (mostly multiple tree-crop households) both produce and buy food, especially in market-oriented areas like Kade. These households can generally afford what they need but often opt for high-status processed foods like instant noodles and aromatic “perfume rice”, which are not necessarily more nutritious. This explains why greater food spending does not always improve nutritional quality, which is consistent with the findings on household dietary diversity scores.

Focus groups also highlighted the growing importance of rice as a staple food. Once a luxury, rice is now increasingly preferred for its affordability and convenience:

“Rice is on the market because it is imported and not so expensive (…) a small amount can feed a lot” *(Focus group Dunkwa, July* *2018).*^2^^2^Quotes may have been edited for clarity and flow without compromising their content. Where mentioned, respondent names are pseudonyms.

“It [rice] is easy to cook, less time-consuming and can be prepared in many forms. (…) [even] without sauce, meat, or fish but with ground pepper or fried egg. You can even omit the egg if you don’t have some” *(Focus group Dunkwa, July* *2018).*

The following quotes reveal shifting dietary patterns, especially among younger generations.

“When we were kids, we ate rice only during Christmas, but now some eat it throughout the year. The children love it and can eat it (…) like we eat our fufu religiously” *(Focus group Kade, September* *2018).*

“My children only want to eat rice or *indomie* (noodles). When you cook *fufu*, they care less, even if they are hungry…” *(Female farmer, focus group Tepa, August 2018).*

While many farmers underestimate the implications of shifting food patterns and increasing market reliance, others are concerned:

“A time may come when we decide to replant the old oil palm farms with food crops if food becomes too expensive” *(Focus group Kade, September 2018).*

The risks are particularly evident in oil palm-dominated areas like Kade and Dunkwa, where declining food-crop land and the inability to intercrop may worsen seasonal food insecurity between January and July.

#### 4.2.4. Food stability

Overall, 58% of households reported difficulty accessing food during June and July. Table 6 shows that significantly higher proportions of landless (76.7%) and single tree-crop households (70.8%) struggled during this period, compared to only 40.6% of multiple tree-crop households (*p* = 0.001). The higher seasonal food insecurity among landless and single tree-crop households is due to limited or no food production during these months and restricted purchasing power to supplement their food supply during food-scarce months.

**Table 6.** Households struggling to obtain food during certain periods of the year per profile.

Profile	Landless households (*n* =30)		Single tree-crop households (*n*=48)		Multiple tree-crop households (*n*= 64)		Absentee households (*n*=26)		Total households (*N* = 168)		*P*-value
	n	%	n	%	n	%	n	%	N	%	
Households experiencing seasonal food insecurity	23	76.7+	34	70.8+	26	40.6-	15	57.7	98	58.3	0.001*

Chi-Square test, X2 (3, *n* = 168) = 15.496, Cramer’s V= 0.304. Test significant at *p* ≤ 0.05. * = statistically significant relationship based on a value adjusted residue. Adjusted residue (AR) > +2 indicates a significantly higher proportion than expected; adjusted residue (AR) < −2indicates a significantly lower proportion than expected.

Source: Survey, 2015; 2017.

Focus group discussions confirmed that from August to December, most farmers are food secure, thanks to harvests of vegetables, fruits (papaya, avocado, and citrus), and wild foods, and income from cocoa and oil palm sales. This allows for regular consumption of *fufu*, a daily staple dish made from cassava, yam, cocoyam, and plantain.

“[During these months,] we eat *fufu* every day. If we don’t, it feels like sleeping on an empty tummy” *(Focus group Dunkwa, July 2018*)

From January to March, mild to moderate food insecurity sets in due to the dry *harmattan* season, which makes soil dry and hampers harvesting of root and tuber crops. Associated windstorms cause plantain damage. Bushfires also threaten crops. From April to July, food scarcity worsens as cassava tubers spoil from leaf regeneration during rains, making them too starchy to be used as food. During these challenging months, wild foods like mangoes, mushrooms, and snails become essential:

“These months can be terrible. Some days, households survive on mangoes alone” *(Focus group Tepa, August 2018).*

“Mango and avocado are abundant from April, and we use them for food and to sell for cash in the cities. However, these fruit trees are disappearing” (*Focus group Kade, September 2018).*

June and July are the most difficult months. Farmers run out of food, and scarcity and high market prices combined with low income capacity make purchasing difficult. Maize- and cassava-based foods like *banku*, *Fanti kenkey, konkonte* and *garri* become more common.

“*Fufu* is still eaten, but spoiled cassava means you end up with too little to feed the whole family” *(Focus group Tepa, August 2018).*

During these months, meals are often meatless or use cheap proteins like salted fish (*koobi*, *momoni*), crabs, grasscutters, or mushrooms.

“We prepare soups with leafy greens and no fish or meat. Sometimes crabs if you farm near water or grasscutters if you are a hunter. Snails are rare. We eat mushrooms sometimes” *(Focus group Kade, September 2018).*

Some mothers skip meals to feed their children during these months. July is even nicknamed *Kitawonsa*—“hold your hands” – symbolising hardship and reliance on others or even theft.

“Sometimes I give the little food to my children, and go to bed hungry” *(Focus group Kade, September 2018).*

**Figure 4.**
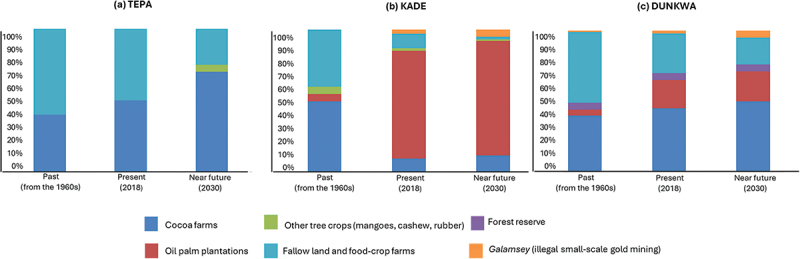
Land uses identified by farmers in the three study areas and their estimated changes through time.

*Source*: Focus groups Tepa, Kade and Dunkwa, July-September 2018.

Land-use change may affect food stability in the long term. Using proportional piling, farmers estimated these changes for the past (since the 1960s), present (2018), and future (up to 2030) (see Figure 4). They observed major shifts from fallow and food-crop land to tree crops, a trend that they anticipate will continue in the future. Cocoa has expanded due to the influx of migrant sharecroppers and oil palm due to outgrower schemes and stable markets.

“Now most land is under cocoa or oil palm. Food-crop farms are scattered. We grow oil palm now instead of food crops—it’s more lucrative than cocoa now” *(Focus group Dunkwa, July 2018).*

(Focus group Dunkwa, July 2018)

“Farming land is getting scarce. Cocoa brings more income than food crops, so we convert all land to it, leaving no land for food production. I intercrop with food crops until the cocoa canopy closes” *(Male farmer, Focus group Kade, September 2018).*

**Figure 5.**
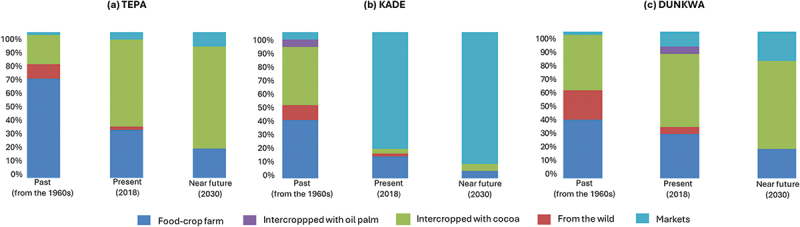
Farmers’ estimated and anticipated changes in food sources across the three study areas.

*Source*: Focus groups Tepa, Kade and Dunkwa, July-September 2018.

These shifts impact food production and sources (Figure 5) and increase market reliance, especially in oil palm-heavy areas like Kade. Farmers express mixed feelings:

“If we don’t change, in the future, we will buy more food than we produce. Many farmers prefer buying over planting food crops themselves. But it will not be like in Accra. Food crops will become available when old cocoa and oil palm farms are replanted and food crops are grown between the seedlings. Food will also come from other places where there is less oil palm” *(Focus group Kade, September* *2018).*

To adapt, farmers increasingly interplant food crops in cocoa farms. Intercropping, a traditional farming practice, has become crucial for food production. In cocoa-dominated Tepa and Dunkwa, intercropping is seen as the main source of future food supply:

“We used to intercrop cassava and vegetables in cocoa farms for up to eight years. Some crops, like bananas or yam (*kokooase bayere*)^3^^3^*Dioscorea* spp. even last longer” *(Focus group Tepa, August* *2018).*

In contrast, intercropping is rarely possible in oil palm farms, reducing food-crop production and dietary diversity in regions like Kade. In these regions, farmers anticipate an increasing reliance on markets for their future food supply (Figure 5):

“Cassava and other root crops affect oil palm because their roots interfere, so the [TOPP] project did not allow planting them. Other food crops were allowed until there was no space for crops to survive among the palms” *(Focus group Dunkwa, July* *2018).*

“Cassava is a very important staple crop to us, and oil palm has reduced its production and affected our diet” *(Focus group Kade, September 2018).*

In conclusion, while HFIAS measures and household dietary diversity scores suggest limited severe food insecurity and adequate dietary diversity, the lived experiences shared in the focus groups revealed a harsher reality, especially in June and July. Farmers noted a shift in diet from traditional *fufu* to rice, reflecting changing preferences and increased market dependence. As cocoa and oil palm expand, food from own production may decline further, especially in oil palm areas. Where cocoa farming prevails, farmers expect intercropping to help offset these losses of food-crop land, but in oil palm zones, reliance on purchased food will likely continue to grow.

### 4.3. What the food security concept does not tell us

#### 4.3.1. Autonomy over production, marketing, and consumption

Survey data, interviews, and focus groups revealed that oil palm farmers involved in outgrower schemes often lose autonomy over production, marketing, and income use (Table 7). While these schemes offer access to oil palm cultivation, they restrict intercropping with root and tuber crops and limit farmers’ decision-making. In contrast, in cocoa-dominated areas like Tepa, households typically choose crops themselves, usually decided by the household head or jointly with the spouse, reflecting local customs.

“We feared that by joining the outgrower project, we would lose access to firewood and food because TOPP would take the land from us. Now food is becoming expensive” (*Interview with Yaa, Dunkwa, January 2017).*

**Table 7.** Decision-making on cash crops, food crops, marketing, and expenditure.

Study district Decision- maker	Kwaebibirem Municipal District, Eastern Region (Kade area) (*n*=51)		Ahafo Ano-North District, Ashanti Region (Tepa area) (*n*=60)		Upper Denkyirah East Municipal Central Region (Dunkwa area) (*n*=57)		Total households		
	n	%	n	%	n	%	N	%	
*On cash crops*									
Household head	21	41.2−	55	91.7+	17	29.8−	93	55.4	0.000*
Spouse	2	3.9	0	0.0	3	5.3	5	3.0	
Head & spouse	3	5.9	3	5.0	5	8.8	11	6.5	
Male (not hh)	0	0.0	2	3.3	1	1.8	3	1.8	
VCC	25	49.0+	0	0.0	31	54.4+	56	33.3	
*On food crops*									
Household head	19	37.3−	47	78.3+	22	38.6−	88	52.4	
Spouse	11	21.6	6	10.0	11	19.3	28	16.7	0.000*
Head & spouse	1	2.0	1	1.7	2	3.5	4	2.4	
Male (not hh)	0	0.0	1	1.7	0	0.0	1	0.6	
VCC	20	39.2+	5	8.3−	22	38.6+	47	28.0	
*On marketing*									
Household head	32	62.7	52	86.7+	22	38.6−	106	63.1	0.000*
Spouse	3	5.9	0	0.0−	5	8.8	8	4.8	
Head & spouse	1	2.0−	1	1.7−	19	33.3+	21	12.5	
Male (not hh)	1	2.0	3	5.0	1	1.8	5	3.0	
VCC	14	27.5+	4	6.7−	10	17.5	28	16.7	
*On spending hh income*									
Household head	37	72.5	51	85+	25	43.9−	113	67.3	0.000*
Spouse	0	0.0	0	0.0	1	1.8	1	0.6	
Head & spouse	14	27.5	6	10.0−	22	38.6+	42	25.0	
Male (not hh)	0	0.0	3	5.0	1	1.8	4	2.4	
VCC	0	0.0	0	0.0−	8	14.0+	8	4.8	

Fisher’s exact test at significant level *p*≤0.05. *: = statistically significant relationships as *p*=0.000. Significant differences based on a value of the adjusted residue (AR). Adjusted residue (AR) >+2 indicates a significantly higher proportion than expected; adjusted residue (AR) < −2 indicates a significantly lower proportion than expected.

Key: hh=household; VCC=value chain collaboration, e.g. in an outgrower scheme.

Farmers in outgrower schemes are also obliged to sell exclusively to the company, even when local buyers offer better prices:

“We are obliged to sell to TOPP. If you try to sell elsewhere, you’re arrested. Prices are higher in the community, but TOPP is paying far less. Also, they take away your farm without compensation if you are unable to maintain it. All the money goes to TOPP” *(Focus group Dunkwa, July 2016).*

“The GOPDC^4^^4^GOPDC is the Ghana Oil Palm Development Corporation. contracts last 25 years. During this time, you can’t sell to anyone else, even if their price is not good—it feels like a monopoly, like you are stuck for 25 years”(Interview with Edward, Kade, February 2017).

Harvest deductions to repay loans and input costs often result in mounting debt:

”TOPP deducts the money they spend on the farm. They take 25% of every harvest plus input and labour costs. Sometimes there’s nothing left” *(Interview with Kwame, Dunkwa, January 2017).*

Farmers also reported losing seed autonomy and being dependent on government-distributed hybrid varieties now. This shift has affected their local varieties as well as their ability to plant what and when they want:

“We no longer use saved or shared seeds. MoFA^5^^5^MoFA is the Ministry of Food and Agriculture. says our varieties are poor and do not yield much. Now we must buy hybrid seeds every planting season” *(Focus group Dunkwa, July* *2018).*

Autonomy is also limited for cocoa farmers without food-crop land and those unable to intercrop. Several rely on labour-for-food or land-for-food arrangements with no control over the food crops they produce, especially landless and single tree-crop households. Under such arrangements, food crops like maize are grown, with harvests divided between farmer and landowner on a fifty-fifty (*abunu*) or one-third-two-thirds basis (*abusa*).

In summary, smallholders value autonomy over what they grow, how they market, and how they spend. Outgrower participation limits control over crop choice, seeds, marketing, and, sometimes, even land. Replacing food-crop land to tree crops further reduces food production, especially affecting landless and single tree-crop farmers. Cocoa farmers turn to sharecropping arrangements to grow food, but oil palm outgrowers often lack such alternatives.

#### 4.3.2. Sustainability of food production from farmers’ perspective^6^6Sustainable tree-crop production is more than the aspects addressed here (see Laven & Boomsma, 2012).

Smallholders across all three study areas expressed concerns about declining food-crop land but were optimistic about sustaining food production through intercropping.

“Our lands, especially those near Kade and Kwae, are now finished. Whatever land is left is fallow lands and very small. The future of food crops lies in replanting old cocoa and oil palm farms and interplanting with food crops” *(Focus group, Kade, September 2018).*

“Usually, when farmers start new cocoa farms, food is abundant because food crops dominate at the early stage of making a cocoa farm. But as the cocoa matures, food becomes scarce since the crops can’t survive under the canopy.” *(Focus group Tepa, August 2018).*

Farmers also raised concerns about *how* food is produced. In all six focus groups, they ranked the use of agrochemicals – especially weedicides – as the top sustainability challenge for crop growth, soil fertility, and pest and disease control:

“Most of us spray weedicides to clear land and control weeds. This has affected mushrooms that used to grow here yearly—even in farms. Now, we must search fallow and forest areas. Snails are gone, too. Even cocoyam, which used to regenerate after clearing fallow land, no longer comes up due to spraying weedicides. We now buy the corms to plant” *(Focus group Dunkwa, July 2018).*

Others highlighted concerns about food safety linked to agrochemicals (Focus group Kade, September 2018). Still, farmers acknowledged that agrochemicals are integral to their farming practices now, making farming easier and boosting production, especially through the use of fertilisers:

“We didn’t use fertiliser on cocoa or oil palm before. But now, without applying fertiliser, cocoa yields nothing. Many of us struggle to afford it” *(Focus group Dunkwa, July 2018).*

Sustainability practices are promoted through various public-private partnerships, often related to certification premiums or community development activities funded by corporate social responsibility projects. However, many farmers find these practices too rigid.

“How can they [TOPP] tell us not to kill snakes, rats, and squirrels eating my oil palm? I fell from a palm tree during harvest after seeing a snake but got nothing for not killing it. Organic fertilisers are expensive, so I buy inorganic fertiliser” *(Focus group Dunkwa, July 2018).*

“Weedicides kill weeds instantly but also kill snails and rats. So, how do soil organisms survive it? After using weedicides for a long time, the soil becomes harder” *(Male participant Tepa Focus group, July 2018).*

In conclusion, while intercropping food crops with tree crops can help sustain food production, food-crop farming alone cannot sustain farmers’ FNS because of low incomes and growing dependence on markets. Despite improving yields, agrochemicals are seen as threats to sustainable farming, soil health, wild food sources, and food safety. Certification-based sustainability efforts mostly involve better-off farmers (see Ataa-Asantewaa, 2023).

## 5. Discussion

### 5.1. A multidimensional approach towards food security

We combined experienced-based food security indicators with food sovereignty dimensions to assess FNS among tree-crop farming households. The findings (see synthesis in Supplementary Material 8) show no statistically significant differences in food availability from own production and dietary diversity scores. However, tree-crop expansion and market orientation affect household profiles’ food access and stability differently, depending on land access, crop type, and farm stage. For instance, landless farmers, single tree-crop households with mature farms, and oil palm outgrowers often face severe seasonal food insecurity due to reduced land for food crops and limited income that constrain access to markets (c.f. Anderman et al., 2014). These households may rely on land-for-food or labour-for-food arrangements with limited benefits and decision-making power. Multiple tree-crop households, in contrast, are significantly more food secure and better able to access food markets.

Several findings align with previous studies. First, our analysis confirmed the multidimensional nature of FNS and the importance of cultural norms and generational shifts in food preferences for smallholders’ FNS (c.f. Noack & Pouw, 2015; Reincke et al., 2018). Second, access to land, farm diversification, and continued household food production remain vital for FNS in areas of commercial crop expansion (c.f. Bymolt et al., 2018; Dompreh et al., 2021; Hashmiu et al., 2022; Ickowitz et al., 2019). Third, consistent with Bymolt et al. (2018), we found that purchasing power is especially crucial during the lean cocoa season when food crops are also scarce. Off-farm income, alongside household food production and market access, was also identified as key to FNS in a study covering 17 countries in sub-Saharan Africa (Frelat et al., 2016). However, while higher incomes can stabilise FNS, they do not always improve dietary diversity, as spending often favours luxury items over nutritious foods (see also Betge et al., 2020; Ickowitz et al., 2019). Fourth, tree-crop type and development stage matter, as food crops like cassava and plantain can be intercropped in young cocoa farms (<5 years) (Schroth & Ruf, 2014) but less in oil palm plantations.

Some findings also nuance or contradict earlier research. First, contrary to Bymolt et al. (2018), our cluster analysis did not identify gender as a defining factor. Though sharecroppers and caretakers are mostly male and absentee farmers more often female, all profiles include both male- and female-headed households (Ataa-Asantewaa, 2023). Second, prior studies argue that tree-crop expansion and commodification reduce food-crop land, with negative effects on household food production (e.g. Anderman et al., 2014; Asubonteng et al., 2018; Asubonteng et al., 2025), especially when women shift to oil palm processing (Vos, 2017). However, more households than expected still grow food. Crop diversification, animal rearing and intercropping support FNS and are particularly vital for low-income households (Aneani et al., 2011; Bymolt et al., 2018; Steenhuijsen Piters et al., 2021; Waha et al., 2018).

Third, our study reinforces calls to integrate food security and food sovereignty (e.g. Byaruhanga & Isgren, 2023; Clapp, 2014; Hopma & Woods, 2014; Jarosz, 2014; Noll & Murdock, 2020), adding empirical evidence to the relationship between FNS and food sovereignty dimensions. For instance, in outgrower schemes, the lack of autonomy over crop choice directly affects food availability, as intercropping food crops is not allowed. Similarly, the inability to choose buyers that offer higher prices reduces outgrowers’ purchasing power and, hence, their economic access to food markets. Food stability may also be undermined if farmers become trapped in cycles of debt.

From a sustainability perspective, the use of agrochemicals in tree-crop cultivation affects all dimensions of FNS. It reduces the availability of wild foods; weakens economic access to food by increasing production costs and eroding farmers’ purchasing power; raises concerns about food safety, an aspect of food utilisation; and undermines the long-term stability of food supply. The growing reliance on market-bought food reinforces this cycle, as it increases the pressure to intensify cash crop production.

Applying a food sovereignty lens also highlights the cultural significance of food and generational shifts in dietary preferences, such as the move from *fufu* to rice. The expansion of tree crops also threatens the availability of *fufu* ingredients like cassava, cocoyam, yam, and plantain, thereby compromising access to culturally appropriate foods. These findings provide complementary insights often overlooked in mainstream food security research and highlight the need for further investigation.

Finally, our multidimensional approach, combining quantitative and qualitative data, offers a comprehensive understanding of FNS among tree-crop farming households. While not always aligned with scientific and expert knowledge, farmers’ lived experiences and local knowledge offer essential and relevant insights for policy and practice.

### 5.2. Study limitations and suggestions for further research

This study had several limitations that merit further investigation. First, selecting communities based on the availability of public transport may have excluded more isolated villages where FNS challenges could be more acute. While our data does not suggest worse outcomes in the two communities without public transport, further research should explore whether FNS differs in less accessible areas.

Second, due to budget constraints, we were unable to conduct multiple visits per year, limiting the study to a cross-sectional snapshot. To minimise bias, data collection took place during the early stages of the dry season (starting in November), when food challenges begin but have not yet peaked (June–July), following guidance by Coates et al. (2007). This timing, along with triangulation from focus group discussions, helped ensure that our findings were neither overly optimistic nor overly pessimistic. Still, reliance on snapshot data and recall periods – 30 days for HFIAS and 48 hours for dietary diversity – limits our ability to capture seasonal changes, price volatility, and daily dietary patterns. Insights from the focus groups suggest that seasonal food insecurity may be more severe than indicated by the quantitative data, although this could be influenced by the timing of the discussions, which began in July – the peak month of seasonal food insecurity. Longitudinal research could offer a more comprehensive understanding of these fluctuations.

Third, caretakers may be underrepresented, as they are not always authorised to speak on behalf of the landowners. Further research should focus on these often-overlooked “invisible farmers”, who are also frequently excluded by extension services and government programmes (Laven & Ataa-Asantewaa, 2025).

Fourth, our data does not allow for a quantitative comparison between cocoa-only and oil palm-only households since most oil palm farmers in our sample also grow cocoa. Given signs of disadvantage among oil palm outgrowers – such as debt accumulation, loss of autonomy of crop choice and marketing, and declining access to food-crop land – further research is needed on their FNS.

Fifth, while we assumed that farmers’ production and sales imply food availability, we lack quantitative data on actual food production to assess whether it meets household needs. This gap is common in food security research. A review of 78 studies by Manikas et al. (2023) found that only one used availability as an indicator, and just two addressed all four FNS dimensions. Most studies relied on experience-based indicators like HFIAS (40%) and dietary diversity scores (44%). This aligns with our approach, but Frelat et al. (2016) convincingly showed the importance of linking food availability to needs and FNS. This is especially relevant given the ongoing decline in food-crop land due to expanding tree crops (e.g. Anderman et al., 2014; Asubonteng et al., 2018).

Sixth, we recommend more research into the impact of labour-for-food and land-for-food arrangements on farmers’ FNS.

Finally, our analysis selectively addressed certain aspects of food sovereignty. Future research should also consider broader aspects of sustainability, self-determination, affordability, and respect for cultural diversity and local knowledge.

## 6. Conclusion

This paper highlighted widespread food and nutrition insecurity (FNS) among cocoa and oil palm farmers, particularly among landless households and those cultivating a single tree crop. Food availability and utilisation did not significantly differ across different household profiles. However, households with higher incomes, such as those with multiple tree crops or absentee owners, experienced more stable FNS during periods of seasonal food scarcity. Conversely, households engaged in oil palm outgrower schemes face restrictions on growing staple crops and accumulating debts, with possible adverse effects on their FNS. The study underscores the importance of diversification of crops, production, and incomes, as well as access to food-crop land and tree-crop incomes for maintaining FNS, especially given the trade-offs between tree-crop and food production.

The findings imply that new approaches are needed to safeguard FNS in tree-crop farming areas. Recommendations are threefold. First, with the decline of food-crop-only farms and shrinking fallow lands, interplanting tree and food crops becomes increasingly vital for subsistence. While intercropping is widespread in the cocoa sector during farm establishment, it is currently not allowed in oil palm outgrower schemes. To support intercropping in oil palm farms, companies could explore alternative planting designs and crop combinations suitable for smallholders.

Second, smallholder policies should promote livelihood diversification across all smallholder groups to enhance income and improve access to food markets. This is particularly important for cocoa farmers, who face seasonal food insecurity in the months leading up to harvest. The study suggests that cultivating multiple tree crops – such as cocoa and oil palm – can significantly improve food security.

Third, concerns about food quality, safety, and affordability were prevalent among smallholder tree-crop farmers. Although the food sovereignty movement has limited traction in Ghana, civil society organisations can help raise awareness about more inclusive and sustainable farming practices. These should draw on farmers’ local knowledge and support grassroots innovation (“innovations from below”) (Laven et al., 2017; Ros-Tonen et al., 2019).

Finally, the study emphasises the importance of integrating cultural values, autonomy, and sustainability into FNS analyses. Incorporating these food sovereignty dimensions allows for a more comprehensive understanding of the multidimensional nature of FNS and can guide efforts to balance market-oriented production with local food needs and sustainable agriculture.

## Supplementary Material

Supplemental Material
