# Supplementary material for: ‘We’re not all the same’—how heterogeneity among smallholder tree-crop farmers in Ghana generates different degrees of food insecurity
Source: NJAS Impact Agric Life Sci. 2025 May 28;97(1):2508143. doi: 10.1080/27685241.2025.2508143 (PMC12327230; doi:10.1080/27685241.2025.2508143)
Supplement: Supplemental Material [file TJLS_A_2508143_SM6858.docx]

**Supplementary Material 1: Focus group protocol related to FNS**

1. *Explore changes in food production, availability, access and consumption caused by farmers’ engagement in commodity production.*
2. **Construct a 10-year timeline** for the landscape to discover changes in the size of forests, food crop farms, cocoa farm areas and oil palm areas (indicate size in approximate percentages of total surface). What do the changes entail? (probing questions: How have food crop farms changed? Discuss when the changes started and why. Extend the timeline into the future. Do you expect changes in the next 10 years, and if so, how and why? Indicate important events that contributed and will contribute to future changes.
3. **Construct a 10-year timeline** to discover changes in food production patterns indicating changes over the period. What do the changes entail? (probing questions: other food items in the past or now? More from the market rather than from own land?). Discuss when the changes started and why. Extend the timeline into the next 10 years. Do they expect further changes, and if so, how and why? Indicate important events that contributed to the changes. Are these changes the same or different for youth/elderly, men/women, and different types of farmers?
4. **Construct a seasonal calendar of food accessibility and consumption** over the 10-year period. What types of foods were accessible to farmers, and by what means? (Farm, market, hunting, wild, ponds? Which time of the year were these foods most available or less? Indicate reasons for availability or absence. What food(s) are consumed by households (list in order of importance)? Does this differ according to tribe, age, gender, or type of farmer? Why? Does this differ across times of the year? Why? Does this pose a challenge, and is the challenge the same or different for youth/elderly, men/women, and different types of farmers? Why? How are these challenges overcome? Do you expect changes in the next 10 years? What reasons may account for these changes?
5. *Explore changes in farmers’ autonomy over resources, production, marketing and consumption caused by engagement in commodity production.*
6. How do farmers get access to land, and under what conditions? How has it changed over the last 10 years (positive or negative or stable)? What accounted for these changes? What determines farm size, crop choice, and who should plant what? Are these observations the same or different for young/elderly, women/men, and different farmers? How do you expect it to change in the next 10 years, and what will account for such changes?
7. How do farmers get access to seeds/planting materials, and under what conditions? How has it changed over the last 10 years? What accounted for these changes? Are these observations the same or different for young/elderly, women/men, and different farmers? How do you expect it to change in the next 10 years, and what will account for such changes?
8. How do farmers get access to farming inputs, and under what conditions? How has it changed over the last 10 years? What accounted for these changes? Are these observations the same or different for young/elderly, women/men, and different farmers? How do you expect it to change in the next 10 years, and what will account for such changes?
9. How do farmers get access to markets, and under what conditions? What produce do they market? How has it changed over the last 10 years? Are these observations the same or different for young/elderly, women/men, and different farmers? How do you expect it to change in the next 10 years, and what will account for such changes?
10. How do farmers get access to capital, and under what conditions? How has it changed over the last 10 years? What accounted for these changes? Are these observations the same or different for young/elderly, women/men, and different farmers? How do you expect it to change in the next 10 years, and what will account for such changes?
11. *C. Explore changes in farm-level sustainability measures due to farmers’ engagement in commodity production.*
12. How do farmers maintain soil fertility and production capacity (list and rank in order of importance)? What are the effects of these measures on the environment (forest, water, soils, and animal species)? How has it changed over the last 10 years, and how do you expect it to change in the next 10 years? (List and rank changes as well)
13. How do farmers manage pests and diseases (list and rank in order of importance)? What are the effects of these measures on the environment (forest, water, soils, and animal species)? How has it changed over the last 10 years, and how do you expect it to change in the next 10 years? (List and rank changes as well)
14. How do farmers control weeds (list and rank in order of importance)? What are the effects of these measures on the environment (forest, water, soils, and animal species)? How has it changed over the last 10 years, and how do you expect it to change in the next 10 years? (List and rank changes as well)
15. How do farmers manage water (list and rank in order of importance)? What are the effects of these measures on the environment (forest, water, soils, and animal species)? How has it changed over the last 10 years, and how do you expect it to change in the next 10 years? (List and rank changes as well)
16. How do farmers manage farm waste (list and rank in order of importance)? What are the effects (s) of these measures on the environment (forest, water, soils, and animal species)? How has it changed over the last 10 years, and how do you expect it to change in the next 10 years? (List and rank changes as well)
17. What types of farming methods do farmers practice? (List and rank in order of importance). What are the effects (s) of practices on the environment (forest, water, soils, and animal species)? How has it changed over the last 10 years, and how do you expect it to change in the next 10 years? (List and rank as well).

**Supplementary Material 2: Food groups used for data entry**

| **Code** | **Food group** | **Description** |
| --- | --- | --- |
| 1 | Cereals | Maize, millet, sorghum and related dishes. |
| 2 | Roots and tubers | Yam, cassava, plantain, cocoyam, taro, potatoes, and related dishes. |
| 3 | Vegetables | All vegetables, local and foreign, known to farmers: cocoyam leaves, *ayoyo* (Corchorus leaves), cassava leaves, *wawa* leaves, onions, tomatoes, garden eggs, okra, pepper, cabbage, carrot, etc. |
| 4 | Fruits | All fruit types: oranges, bananas, mangoes, avocado, pawpaw, etc. |
| 5 | Meat | Chicken, turkey, duck, birds, beef, pork, mutton, lamb, wild game, etc. |
| 6 | Fish | Fresh, dried, salted shellfish or seafood. |
| 7 | Egg | Eggs from poultry or other birds. |
| 8 | Legumes, nuts and seeds | Beans, peas, peanuts, soybeans, etc. |
| 9 | Dairy products | Milk, yoghurt or other milk products |
| 10 | Oils and fats | Palm oil, olive oil and other cooking oils |
| 11 | Sugars | Sugar cubes, powder, sweeteners |
| 12 | Beverages | Tea and coffee, alcohol and non-alcoholic drinks. |

*Source:* FANTA (2007).

**Supplementary Material 3: The iterative clustering process and resulting variables**

The most critical decision in a clustering process is selecting the appropriate characteristics or variables. This choice depends on both the purpose of clustering and the expected quality of the cluster solution (Mooi & Sarstedt, 2011). To ensure high-quality clusters, the selected variables need to distinguish groups clearly and explain as many differences in the dependent variables as possible. Additionally, the number of variables has to be reasonable relative to the sample size. While there is no universally accepted minimum, it is generally recommended that the sample size be at least ten times the number of variables used to ensure sufficient cluster quality (Mooi & Sarstedt, 2011).

For this study, maintaining an appropriate ratio between the largest and smallest clusters was also crucial, as each cluster required a substantial number of respondents for further statistical analysis. A minimum sample size of 30 per group is considered ideal for testing statistically significant differences among groups, with the ratio between the largest and smallest clusters not exceeding 1.5 (Stevens, 2009, p. 239; Pallant, 2016).

Based on the sample size of 168 and the recommendation to limit the number of variables to one-tenth of this size, 17 variables were initially selected: gender, origin, and age of the household head; household composition; formal education level; tree-crop farm size (hectares); farming intensity; food production; livestock production; diversity of tree crops; income; labour; access to government programmes; access to extension services; access to value-chain collaborations; presence of a spouse; and spouse’s occupation.

Of these, nine were considered independent variables to explain variations in the dependent variables: gender, age, household size, education level, total land size, farming intensity, diversity of tree crops, and the origin and occupation of the spouse. However, due to poor data quality, the origin of the household head and the spouse’s occupation were excluded. This left seven variables for cluster analysis, which were subjected to an iterative clustering process to determine the optimal cluster solution.

Initially, these seven variables produced four clusters of fair quality (0.3), with a largest-to-smallest cluster ratio of 1.55. The variables ranked in order of importance were the gender of the household head, farming intensity, diversity of tree crops, tree-crop farm size (ha), education level, household size, and age of the household head.

To improve the cluster solution, the least important variable was removed iteratively until the outcome met the following criteria: an importance value of at least 0.5 and a cluster size ratio of no more than 1.5. Removing age did not change the number of clusters, cluster quality, or distance ratio. Removing household size as the next least important variable also retained the four clusters, with an improved ratio of 1.50 and fair quality (0.4). The same applied when education was removed.

After removing education, the four remaining variables—gender of the household head, farming intensity, diversity of tree crops, and tree-crop farm size (ha)—produced the same four clusters but with improved quality (0.6) and a 1.45 cluster size ratio. While this was reasonable, tree-crop farm size (ha) emerged as the least significant variable, showing no clear differentiation among groups. Removing tree-crop farm size (ha) further improved the cluster quality to 1.0 but resulted in eight clusters with a largest-to-smallest ratio of 6.0, which was not suitable for the study and was therefore rejected.

Thus, the final cluster solution retained four key variables—gender of the household head, farming intensity, diversity of tree crops, and tree-crop farm size (ha)—which generated four well-differentiated clusters suitable for the study.

**Supplementary Material 4: Seasonal calendar sheets per district**

**Farming and seasonal food calendar, Kwaebibirem Municipal**

| **Month**  **Food/activity** | **J** | **F** | **M** | **A** | **M** | **J** | **J** | **A** | **S** | **O** | **N** | **D** |
| --- | --- | --- | --- | --- | --- | --- | --- | --- | --- | --- | --- | --- |
| Land preparation/  farm maintenance | Clear­ing of land by slashing | Spray the cleared land with weedi­cides to avoid burning |  |  |  |  |  |  |  |  |  | Land prepara-tion can start here into the new year. |
| Planting |  | Plant *Abrokan* (first maize). Oil palm seedlings can also survive planting in March, but only if they have 4-5 leaves. | | Plant food crops – plan­tain, maize, coco­yam, yams, vegeta­bles from the end of March | Plant oil palm and co­coa with food crops. Food crops help cocoa seedlings to grow. Cassava cannot go together with oil palms as they interfere with the roots. | |  |  | Full-sun maize (*Awia fuo* = sun farm) cannot be mixed with other crops; “it won’t yield”. |  |  |  |
| Harvesting |  |  |  |  | *Abrokan* (early maturing maize) is ready for harvest. It is usually sold fresh, boiled or grilled on the market. | Maize should be har-vested in early June; else, the June-July rains will cause it to ger-minate. |  |  |  |  |  | Can harvest cassava, coco­yam and some yams during Decem­ber. |
| Coping with food shortages |  |  |  |  | These are the most difficult months. It rains a lot; people cannot go to the farms. Food crops get rotten; cassava regenerates new leaves and spoils. Eating *fufu* is challenging, and it has been replaced with *konkonte*, *banku*, and rice. Rice is mainly for children who do not like *fufu*. | | |  |  |  | Vegetables are scarce now, but immature pawpaw (papaya) may be available starting in November and is used to replace tomatoes. | |
| Cocoa and oil palm | There are no yields from the oil palm in these months.  Cocoa money is al­ready spent, and no lean cocoa season any­more | | Main oil palm season starts |  |  |  |  | The cocoa season opens (small harvest). The main cocoa season has moved from August to Octoberdue to hybrid varieties |  | Main cocoa season peaks. |  | Oil palm yields decline dramati-cally, and palm oil be­comes expen­sive.  Cocoa harvest starts declin­ing. |
| Food crops (tubers, roots, suckers) | We eat *fufu* every day. Also, *ampesi,* but the children don’t like *ampesi or fufu*. They like rice and *banku*. | | | | People eat *konkonte, gari, banku*, rice. Rice and *banku* are not too expensive to prepare. A small amount can feed a lot of people. | | | There is abundant cash and food in these months after shortages in the prior 3 months. | | | Food starts getting scarce. People ran out of money in December due to Christmas. Luxurious food during Christmas. | |
| Vegetables | Dry sea­son veg­etable farming starts from Decem­ber but requires irrigat­ion. |  | Dry-season vegeta­bles are available (toma­toes, okra, garden eggs).  Leafy vegetables (e.g. cocoyam leaves) are abundant from March when the rains start. | | Immature *pawpaw* (papaya) fruits are used to replace toma­toes and garden eggs. Mushrooms are eaten once in a while. | | | Vegetables intercropped with food crops are available. | | | | Vegeta­bles are ex­pensive, notably toma­toes. The salad is made with carrots, lettuce, and cabbage bought in town. There is no own prod­uction of these crops. |
| Fruits |  |  | Wild mangoes and avocados are abun­dant. | |  | | | *Pawpaw*, avocado, and oranges season. | | | | Fruits are becom­ing scarce. |
| Meat and fish | Fish is eaten throughout the year. Cold-store chicken is bought when there is money. Home-reared chickens are also eaten. | | | | Some eat without fish or meat. Leafy vege­table soups prevail. Those with water near the farm also get crabs. Hunters can get grasscut­ters. | | | No hunting (repro- duc­tion pe­riod). |  |  |  | Chicken and rice for the festive season. |

*Source:* Focus groups in Kwaebibirem Municipal, 2018.

**Farming and food seasonal calendar, Upper Denkyira East Municipal**

| **Month**  **Food/activity** | **J** | **F** | **M** | **A** | **M** | **J** | **J** | **A** | **S** | **O** | **N** | **D** |
| --- | --- | --- | --- | --- | --- | --- | --- | --- | --- | --- | --- | --- |
| Land preparation/  farm maintenance | Land clearing |  |  |  | Weeding mostly. Heavy rains these months are not so good for going to the farm. | | |  |  |  |  | Land clearing |
|  | Apply liquid fertiliser and agrochemicals for hybrid cocoa. Without these, there will be pests and no yields. Rains are ‘slow’, giving fertiliser time to dissolve and not wash away. | | | |  |  |  | Apply fertiliser as rains decline for Tetteh Quarshie co­coa, but it can still yield without it. | |  |  |  |
| Harvesting | Food is available on the farm but is difficult to harvest due to dry soil. | | | | The food we don’t produce is expensive throughout the year, but during these 3 months, what we produce becomes expensive because it becomes scarce. | | |  |  |  | Harmattan starts in late November. Dry soil affects the harvesting of food crops. Farm work becomes less, and we go less to the farm. | |
| Coping with food shortages | Start using stored maize, but it gets used up before June. | | | | Eat *banku, gari, konkonte*, rice*. Fufu* eating becomes a challenge. The quantity and quality of food in June-July are worse. However, they say green vegetables are good, and we eat them. | | |  |  |  |  |  |
| Cocoa and oil palm |  | Cocoa lean season until March. | The oil palm main season starts. | |  |  |  | Oil palm yields start de­clining in March. |  | The main cocoa season starts and peaks by late December or January. | | |
| Food crops (tubers, roots, suckers) | Plant dry sea­son maize. | Some plants early yielding cassava to be harvest­ed in May.  Rice is planted from late Feb­ruary to March | Planting maize and other food crops. | |  | Cassava starts rotting or spoiling.  Plantain ex­pensive  Going to the farm is often disrupted by heavy rains. | | Early yielding cas­sava available –usually *Ampesi* cas­sava  Rice is available from late July-August | | Food crops available from Au­gust but hard to harvest from December to February | | |
| Fruits | One may find pa­paya, oranges, and some man­goes but mang­oes are uncom­mon. | Water-melons from the savan­nah areas on the mar­ket in town till late May. | Fruits from the wild and on-farm fruit trees, man­goes and avoca­dos. | The first avocado from the wild and the farm is available. | |  |  | Papaya, avo­cado, and oranges are readily availa­ble | | | |  |
| Vegetables | Dry sea­son veg­etable farming | Fresh foreign vegetables are available on the market in towns, and all-season carrots, cucumber, lettuce, cab­bage, and spring onions. Local dry-season vegetables such as tomatoes, okra, and garden eggs are available starting in March but are expensive. | | | Cocoyam leaves, *Duko* (made from wild green leafy vegetables, without meat or fish). | | | Normal season vegetables are available. They are not expensive; everyone has them on their farm except onions. | | | | Vegeta­bles, especially toma­toes, are expensive due to the dry season and Christ­mas. |
| Meat and fish | Eat mostly fresh fish from cold stores and dry and salted fish from the market till early June.  We get mushrooms with the in­con­sistent rain show­ers from February through April. We often use it as meat. If you want to store it, you smoke it; otherwise, it rots within two days. Most prefer to sell mushrooms because they fetch a huge amount of money at a time like this.  Hunt­ing is al­lowed from November till July. No hunting in August | | | | The worst month for meat or fish. We normally eat without meat or fish because of a lack of money. Snails and crabs are important. Snails come out because of the rain. | | | Fresh, dry and salted fish is often eaten. Once in a while, cold store chicken (*ofa da mpam*) or cow intes­tines (when you want to spend money) | | | | Own chicken, goat, and sheep, or from a cold store. Cow meat from commu­nal kill. |

*Source:* Focus groups in Upper Denkyira East Municipal, 2018.

**Farming and food seasonal calendar, Ahafo-Ano North District**

| **Month**  **Food/activity** | **J** | **F** | **M** | **A** | **M** | **J** | **J** | **A** | **S** | **O** | **N** | **D** |
| --- | --- | --- | --- | --- | --- | --- | --- | --- | --- | --- | --- | --- |
| Land preparation/  farm maintenance | Land preparation | |  |  | Weeding, with much use of weedi­cides. Meals are consumed at home because there is no food on the farm to prepare there. | | |  |  | Prepare land for dry sea­son vegeta­bles. |  | Land prepa­ration starts and continues to Jan-Feb. |
| Planting |  | Plant early maize. |  | Plant food crops. |  |  |  |  |  | Dry- season vege­tables |  |  |
| Cocoa | Money from the main cocoa season finishes here. | Cocoa lean season | Lean cocoa season money is used up by May to make new farms, pay school fees, and feed. | | |  |  |  |  | Cocoa main season. Money is around until January. |  | Most cocoa money is used up for the festive season. |
| Food crops (tubers, roots, suckers) | There is food, but the dry season makes harvesting difficult.  Plantain is abundant and cheap; selling makes no sense.  Use of stored maize.  Fufu is still common. | | | Plantain starts getting scarce and then expensive.  *Kokoase* yams (yams growing under cocoa trees) are available.  Stored maize is coming to an end and is becoming expensive.  Maize food is often eaten in this area, such as *kenkey*, *banku*, and f*ante* *kenkey*. Rice and *konkonte* are also eaten a lot – *konkonte* feels like *fufu,* which is less available in June and July because the cassava is mostly spoiled. | | | | Early maize, early cassava, cocoyam and plantain become available.  *Fufu* can be eaten almost daily and, in some homes, even twice daily until June-July. | | | | Most food crops are available. |
| Vegetables | Pepper and garden eggs from the farm are available until it be­comes too dry. |  |  |  | Leafy vegetables are abundant in the rainy season: cocoyam leaves (*kontomire*), jute leaves (*ayoyo*)*,* water leaves (*bokoboko*), and the edible fresh leaves (*wawa nkon*) from the Wawa tree (*Triplochiton scleroxylon)* used in place of the vegetable okra in soups or stews. Garden eggs are expensive in May-June. | | | Vegeta­bles avail­able. |  | Dry- season plant­ing of cab­bage, toma­toes |  | Some dry-sea­son vegeta­bles are avail­able until January but expensive. |
| Fruits | Watermelon from the North is on the market.  In Tepa, all types of fruits are available on the market all year round: apples, mango, bananas and even pears. However, people cannot afford it or won’t spend money on fruits that they can get for free during their season. They only buy them if a doctor recommends eating them. | | Wild mangoes are available in March and April.  Avocado also becomes available. | |  |  |  | *Pawpaw* comes and vanishes with the food crops.  Avocado is available again. | | | | Watermelon is available from December to March. It is a dry-sea­son crop from the savan­nah ar­eas in the North. |
| Meat and fish | Cold-store fish, dry and salted (*koobi* and *momoni*), is available from the market all year round. During festive seasons, home-raised chickens are killed, and poultry is bought when products are sold in the villages. Sometimes meat is hardly eaten except at Christmas. The children eat meat because they buy food (rice) at school from food vendors who use fish, meat, and eggs. Households eat mostly vegetable soups without meat during June and July. Those raising chickens can use it, but not for two months. Hunting by males can be a source of meat except for August-September when people are not supposed to hunt (reproduction time). | | | | | | | | | | | |

*Source:* Focus groups in Ahafo Ano North District, 2018.

**Supplementary Material 5: Key characteristics per household profile**

| **Household**  **profile**  **Variable** | **Landless households**  **(n= 30, 17.9%)** | **Single tree-crop households**  **(n =48, 28.6%)** | **Multiple tree-crop households**  **(n= 64, 38.1%)** | **Absentee households**  **(n=26, 15.5%)** |
| --- | --- | --- | --- | --- |
| Average age of hh^i^ head (years) | 43.03^a^ yrs, SD 9.70  Median 39  Min 28, Max 65 | 51.79^ab^ yrs, SD 11.55  Median 52  Min 28, Max 75 | 55.81^b^ yrs, SD 9.21  Median 54.50  Min 37, Max 76 | 67.42^bc^ yrs, SD 17.34  Median 70.00  Min 27, Max 100 |
| Average hh size (no. of hh. members) | 7.37, SD 4.82  Median 7.0  Min 1, Max 20 | 6.81, SD 3.47  Median 6.0  Min 2, Max 16 | 8.84, SD 5.73  Median 7.0  Min 2, Max 32 | 7.92, SD 4.10  Median 8.0  Min 2, Max 18 |
| Gender of hh head  proportions (%) | Male 76.7%  Female 23.3% | Male 79.2%  Female 20.8% | Male 89.1%  Female 10.9% | Male 65.4%  Female 34.6% |
| Education of hh head^ii^ | None 46.7%  < Basic 16.6%  Basic 36.7  SHS 0.0%  Tertiary 0.0% | None 29.1  < Basic 16.7%  Basic 43.8%  SHS 4.2%  Tertiary 6.2% | None 14.1  < Basic 17.2%  Basic 56.3%  SHS 6.3%  Tertiary 6.3% | None 30.7  < Basic 15.4%  Basic 38.5%  SHS 7.7 %  Tertiary 7.7% |
| Average tree-crop farm size (ha) | 4.50^a^ ha, SD 3.94  Median 3.30 ha  Min 1.0, Max 21.0 | 4.77^a^ ha, SD 3.55 ha  Median 4 ha  Min 0.8, Max 18.40 | 7.52^b^ ha, SD 4.98 ha  Median 6 ha  Min 0.80, Max 23.0 | 6.13^a^ ha, SD 9.0 ha  Median 3.30 ha  Min 1.0, Max 44 |
| Type of tree crop farm (% of hhs) | Cocoa 100% | Cocoa 91.7%  Oil palm 8.3% | Cocoa & oil palm 100%; Other tree crops 26.6% | Cocoa 50%  Oil palm 15.4%  Both 34.6% |
| Location (% of hhs) | Dunkwa 46.7%  Kade 0.0%  Tepa 53.3% | Dunkwa 27.0%  Kade 18.8%  Tepa 54.2% | Dunkwa 32.8%  Kade 51.6%  Tepa 15.6% | Dunkwa 34.6%  Kade 34.6%  Tepa 30.8% |
| Average annual gross hh income^iii^ (USD) | 686.61^a^, SD 662.24 Median 556.80  Min 22.30  Max 2,929.5 | 1,245.02^b^, SD 918.92, Median 1,082.75 Min 27.80  Max 3,610.3 | 2,035.47^c^  SD 1,410.50  Median 1,562  Min 118.70  Max 6,404.10 | 2,082.32^bc^  SD 2,064.42  Median 1,388.60  Min 222.20  Max 7,809.90 |
| Proportion farm/non-farm income^iv^ (%) | Farm income 81%  Non-farm 19% | Farm income 86%  Non-farm 14% | Farm income 86%  Non-farm 14% | Farm income 80%  Non-farm 20% |

^i^ hhs=households. ^ii =^ Basic=primary (6 yrs.) + JHS (3 yrs); JHS=junior high school; SHS=senior high school). ^iii^ Based on n=162 as 6 had missing data. ^iv^ Based on n=101 households with non-farm income. Averages that do not share the same superscript letter differ significantly at P ≤ 0.05 (Kruskal-Wallis one-way analysis of variance). Groups sharing at least one letter are not significantly different. *Source*: Survey 2015-2017.

**Supplementary Material 6 Harvested food crops reported by the households in the 12 months preceding the survey**

| **Profile**  **Food crops harvested** | **Landless farming households**  **(n =23)^a^** | | **Single tree-crop households (n=40)^a^** | | **Multiple tree-crop households**  **(n= 53)^a^** | | **Absentee households (n=20)^a^** | | **Total households**  **(N=136)** | | **P-value** |
| --- | --- | --- | --- | --- | --- | --- | --- | --- | --- | --- | --- |
|  | **n** | **%** | **n** | **%** | **n** | **%** | **n** | **%** | **n** | **%** |  |
| Plantain | 22 | 95.7 | 38 | 95.0 | 47 | 88.7 | 18 | 90.0 | 125 | 91.9 | 0.701 |
| Cassava | 19 | 82.6 | 31 | 77.5 | 44 | 83.0 | 19 | 95.0 | 113 | 83.1 | 0.416 |
| Cocoyam | 15 | 65.2 | 28 | 70.0 | 27 | 50.9 | 12 | 60.0 | 82 | 60.3 | 0.301 |
| Maize | 5 | 21.7 | 8 | 20.0 | 20 | 37.7 | 8 | 40.0 | 41 | 30.1 | 0.167 |
| Rice | 1 | 4.3 | 1 | 2.5 | 3 | 5.7 | 0 | 0 | 5 | 3.7 | 0.861 |
| Vegetables | 13 | 56.5 | 23 | 57.5 | 27 | 50.9 | 13 | 65.0 | 76 | 55.9 | 0.762 |
| **Diversity** |  |  |  |  |  |  |  |  |  |  |  |
| Min | 1.00 | | 1.00 | | 1.00 | | 1.00 | | 1.00 | |  |
| Max | 6.00 | | 6.00 | | 6.00 | | 5.00 | | 6.00 | |  |
| Mean | 3.26 | | 3.23 | | 3.17 | | 3.50 | | 3.25 | |  |
| SD | 1.39 | | 1.25 | | 1.30 | | 1.10 | | 1.26 | |  |
| Median | 3.00 | | 3.50 | | 3.00 | | 4.00 | | 3.00 | |  |

^a^ n refers to the number of households in the profile growing food; Chi-Square test where minimum cell frequency is 5 or higher; Fisher’s exact test where minimum cell frequency is less than 5. Test significant at p≤0.05.

Crop diversity was measured using Kruskal-Wallis one-way analysis of variance; means significant at p≤ 0.05. P-value 0.733.

*Source*: Survey, 2015; 2017.

**Supplementary Material 7** **Farmers’ perceptions of food (in)security across self-identified profiles**

| Self-identified profile  & cluster equivalent  FNS dimension | Caretakers  (landless farm HHs) | Emerging tree-crop farmers/ sharecroppers (years 1-2) | Tree-crop farm owners  (single tree-crop HHs | Entrepreneurial farmers (‘rich farmers’)  (multiple tree-crop HHs) | Absentee farmers |
| --- | --- | --- | --- | --- | --- |
| *Availability* |  |  |  |  |  |
| - Food from the wild | x |  |  |  |  |
| - Labour-for-food | x | x | x |  |  |
| - Food-crop farm | x | x | x | x | x |
| - Market |  | x | x | x | x |
| *Access* |  |  |  |  |  |
| - Food from the wild | x | x |  |  |  |
| - Labour-for-food | x (~50%)^a^ | x | x |  |  |
| - Food abunu/abusa | x |  | x |  |  |
| - Land-for-food abunu/abusa |  |  |  |  | x |
| - Food-crop farm |  |  | x |  | x |
| - Marketb | x (~20%) | x (~50%) | x (~20%) | x (~50%) | x (~50%) |
| *Utilisation* |  |  |  |  |  |
| Frequency of meals |  |  |  |  |  |
| - May not eat three meals a day | x | x |  |  |  |
| - Can afford three meals a day |  |  | x | x | x |
| Dietary diversity |  |  |  |  |  |
| - May sometimes eat without fish or meat | x | x |  |  |  |
| - Eat meat often |  |  |  | x | x |
| - Eat more food from the wild (bushmeat, fish, crabs, mushrooms) | x | x |  |  |  |
| - Limited dietary diversity: focus on maize, local rice, cassava foods, leafy vegetables; fruits only when in season) | x | x | x |  | *(table continues)* |
| - More fruits and vegetables (e.g. including fruits and vegetables from the farm, wild, also from the market) |  |  |  | x |  |
| *Preferences* |  |  |  |  |  |
| - May aim for a balanced diet |  |  |  | x | x |
| - May overlook fruits |  | x | x |  |  |
| - Eat luxury food often (perfume rice, instant noodles (*indomie*), meat) |  |  |  | x | x |

^a^ Percentages are the participants’ estimates and have no absolute value.

^b^ Mainly for meat, perfume rice, tuna, etc.

*Source:* Focus groups at Tepa, Kade, and Dunkwa, 2018.

**Supplementary Material 8 Synthesis of findings related to FNS per profile**

| **Profile**  **FNS/**  **food**  **sovereignty indicators** | **Landless households**  **(n=30)** | **Single tree-crop households**  **(n=48)** | **Multiple tree-crop households**  **(n=64)** | **Absentee households**  **(n= 26)** | **Observations** |
| --- | --- | --- | --- | --- | --- |
| **Food availability** | All produced food (100%). Many rely on labour/land-for-food arrangements and depend more than other groups on wild foods. | The majority produced food (96%); reliance on labour/land-for-food arrangements during food-scarce months. | The majority produced food (91%); relatively less intercropping due to mature tree farms. | The majority produced food (89%). Many (39%) make food-crop farms when stepping back from tree-crop farm management. | Most households produce food (94%, mainly through intercropping (64%). No statistically significant differences across profiles. |
| **Food access/Household Food Insecurity Access Scale (HFIAS)** | A statistically significant lower proportion (30%) can buy food from markets and is food secure based on HFIAS. Increasing dependence on market-bought food is unfavourable for them. | 44% can buy food from markets, and is food secure based on HFIAS. | The proportion of households that can buy food from the market (65%) and are food secure based on HFIAS (66%) is significantly higher than other profiles. | 58% can buy food from markets, and 46% are food secure based on HFIAS. | Overall, the majority (51%) reported the ability to buy food from food markets. Less than half (47%) of all respondents are food secure throughout the year. Statistically significant differences across profiles. |
| **Food utilisation/ Household Dietary Diversity Score (HDDS)** | Mean HDDS of 8.23. | Mean HDDS of 8.44. | Mean HDDS of 7.94. | Mean HDDS of 7.24. | All profiles have adequate dietary diversity with no statistically significant differences.  *(continues)* |

| **Food stability** | A significantly higher proportion (77%) struggle to obtain food during certain times of the year. | A significantly higher proportion (71%) struggle to obtain food during certain times of the year. | A significantly lower proportion (41%) struggles to obtain food during certain times of the year. | An expected proportion (58%) struggle to obtain food during certain times of the year. | Of all respondents, 58% struggled to obtain food during June-July. |
| --- | --- | --- | --- | --- | --- |
| **Autonomy over production, marketing, spending** | Limited autonomy over production and marketing as they are not the owners of the farm. | As farm owners, they have autonomy over production, marketing, and spending, but a debt relationship with the buyer may exist. | Great autonomy over spending and crop and buyer choice unless involved in an oil palm outgrower scheme. | As land/farm owners, they decide which crops to plant and where to market. Make autonomous decisions on expenditures. | Landless farming households (sharecroppers and caretakers) and households in outgrower schemes have the least autonomy, which undermines food availability from household production. |
| **Culturally appropriate and preferred food** | Seasonal food insecurity affects the consumption of preferred food. | Seasonal food insecurity affects the consumption of preferred food. | Higher purchasing power may lead to the consumption of less nutritious food with higher status. | The ability to buy food implies no need to compromise on preferred food during food-scarce months. | Except for multiple tree-crop households, seasonal food insecurity affects the consumption of preferred food.  All profiles see a shift from the traditional dish *fufu* to rice in younger generations.  Tree-crop expansion and the use of agrochemicals affect the availability of wild foods used in traditional dishes. |
| **Sustainable food production** | The expansion of tree crops undermines food production and increases dependence on food markets.  Intercropping is key to maintaining food production.  Overall concern about agrochemical use (notably weedicides) and effects on biodiversity, wild foods availability, and food safety. | | | | |
